# Factors associated with mortality in patients with cardiovascular diseases affected by COVID-19: a cross-sectional study

**DOI:** 10.1590/1518-8345.7609.4551

**Published:** 2025-05-19

**Authors:** Mariane Cardoso Carvalho, Flávia Emília Cavalcante Valença Fernandes, Matheus Vargas dos Santos Almeida, Joebson Maurilio Alves dos Santos, Simone Coelho Amestoy, Rosana Alves de Melo

**Affiliations:** 1 Universidade de Pernambuco, Campus Petrolina, Petrolina, PE, Brazil; 2 Scholarship holder at the Conselho Nacional de Desenvolvimento Científico e Tecnológico (CNPq), Brazil; 3 Universidade de Pernambuco, Faculdade de Administração e Direito de Pernambuco, Recife, PE, Brazil; 4 Universidade Federal do Vale do São Francisco, Departamento de Enfermagem, Petrolina, PE, Brazil

**Keywords:** COVID-19, Mortality, Cardiovascular Diseases, Risk Factors, Hospitalization, Notification

## Abstract

to analyze the factors associated with mortality in patients with cardiovascular disease affected by Coronavirus Disease-2019.

this was a cross-sectional study using data from the monitoring of notifications during the pandemic. The sample included cases with cardiovascular comorbidity and clinical outcomes. The dependent variable was the progression of the case to death. Associations were tested using the binary logistic regression method, using the Odds Ratio.

the prevalence was female (50.6%), elderly (71.1%), self-declared non-white (71.0%), with multiple comorbidities associated with cardiovascular disease (60.3%), diabetes being the main one (44.8%). The study suggests that patients who were men (OR 1.13; p = 0.028), elderly (OR 2.57; p = 0.000), self-declared white (OR 1.71; p = 0.000), and had multiple comorbidities (OR 1.70; p = 0.000) were associated with a greater chance of death.

the factors associated with a higher chance of death were related to gender, age group, and the presence of comorbidities, showing the vulnerability of this population to infection.

## Highlights

**(1)** Identification of the clinical and demographic profile of patients with CVD affected by COVID-19. **(2)** Mortality-related Factors were sex, age group, race/color, and comorbidities. **(3)** Men, the elderly, and self-declared whites had a higher chance of death. **(4)** The presence of multiple comorbidities associated with CVD increased the chance of death. **(5)** Hospitalization was associated with a lower chance of death.

## Introduction

Cardiovascular Diseases (CVD) are the leading cause of morbidity and mortality in the world and represents a risk to the population, as it is estimated that 17.9 million people died from it in 2019, equivalent to 32% of all global deaths ^( 1 )^. Severe Acute Respiratory Syndrome Coronavirus 2 (SARS-CoV-2), which triggers Coronavirus Disease-2019 (COVID-19), is directly associated with the cardiovascular system since the virus infects host cells through the Angiotensin Converting Enzyme 2 (ACE2) receptors present in various organs, and is highly expressed, especially in the heart and alveolar epithelial cells in the lungs ^( 2 - 4 )^.

The binding of the SARS-CoV-2 Spike protein to the ACE2 protein is followed by virus endocytosis and subsequent viral replication, which causes downregulation of ACE2 ^( 3 )^, and results in respiratory symptoms that become more severe in the presence of CVD, as ACE2 is highly expressed in these patients compared to healthy individuals ^( 2 , 4 )^. As a result, the cardiovascular clinical manifestations of COVID-19 mainly include acute cardiac injury, acute myocardial infarction, myocarditis, arrhythmia, heart failure, venous and/or pulmonary thromboembolism and shock ^( 5 )^.

In addition, in patients with hypertension, coronary heart disease, and other cardiomyopathies, the viral disease can further damage myocardial cells through direct damage caused by the virus, systemic inflammatory responses, destabilized coronary plaque, and aggravated hypoxia. These aspects make these patients more likely to suffer myocardial damage after COVID-19 infection and have a higher risk of death ^( 6 )^. Therefore, patients with underlying CVD, especially the elderly, are susceptible to greater risks of adverse outcomes and death during severe inflammatory responses to COVID-19 than younger, healthier individuals ^( 7 )^.

Heart damage is a common condition among patients hospitalized with COVID-19. It is associated with a higher risk of in-hospital mortality, more specifically in patients with pre-existing CVD, since according to a study carried out in Wuhan/China, approximately 30% and 60% of patients who had heart damage had a history of coronary heart disease and hypertension, respectively ^( 8 )^.

Similarly, a Brazilian study carried out in Rio de Janeiro showed that the incidence of myocardial injury in patients admitted to the Intensive Care Unit (ICU) with a confirmed diagnosis of COVID-19 was 36% of the sample, in which systemic arterial hypertension and Body Mass Index (BMI) were independent risk predictors ^( 9 )^.

The various studies carried out to identify the clinical and/or demographic characteristics that may be associated with the mortality of these patients have shown the influence of demographic variables, such as age, sex, and ethnicity ^( 10 - 11 )^, and clinical factors, such as the presence of comorbidities ^( 12 - 13 )^, as risk factors for the greater severity and mortality of the cases. In the UK, an observational cohort study of 20,133 patients showed that factors such as male sex, advanced age, and the presence of comorbidities were strong predictors of in-hospital mortality ^( 14 )^.

Thus, considering the health emergency that SARS-CoV-2 represents, and the implications caused in patients with CVD, it is necessary to carry out studies aimed at identifying the factors associated with COVID-19 mortality in this vulnerable group, for the production of scientific knowledge, since this knowledge can help to carry out an adequate screening of patients at risk, in addition to making it possible to create subsidies for the adoption of measures and strategies aimed at better control of the disease and prevention of the disease.

Therefore, this study aims to analyze the factors associated with mortality in patients with CVD affected by COVID-19.

## Method

### Study design

This is a cross-sectional analytical study using a database of COVID-19 notifications in the state of Pernambuco.

### Study site

The study was conducted in the state of Pernambuco, a Brazilian state located in the Northeast Region of the country, with a territorial area of 98,067.877 km² and an estimated population of 9,674,793 people in 2021 ^( 15 )^.

### Population and sample

The study population consisted of confirmed COVID-19 cases notified in the state of Pernambuco between March 2020 and December 2022, with patients with any cardiovascular comorbidity recorded in the notification. The total number of cases was 17,522. The sample included cases with information on the clinical outcome, i.e., the case’s progression to death or recovery. The exclusion criteria were cases where clinical and demographic information was missing or inconsistent. Based on these criteria, the final sample consisted of 6,704 patients.

### Study variables

The dependent variable was death as the clinical outcome, while the independent variables were: sex (male/female), age group (elderly or non-elderly), race/color (white and non-white), the presence of multiple comorbidities (yes/no), the classification of these comorbidities which were associated or not with previous CVD, such as associated diabetes (yes/no), associated chronic kidney disease (yes/no), associated respiratory disease (yes/no), associated overweight/obesity (yes/no) and, finally, the need for hospitalization (yes/no).

Elderly patients were considered to be aged 60 or over, according to the Statute of the Elderly Person ^( 16 )^, and non-white patients were considered to be black, brown, yellow, or indigenous.

### Data collection

The information was collected through the “COVID IN DATA” platform, a database for monitoring COVID-19 notifications in the state of Pernambuco, made available by the Planning and Management Secretariat (SEPLAG) in conjunction with the State Health Secretariat (SHS) and the Pernambuco State Information Technology Agency (STI) ^( 17 )^. The database was collected and built between December 2022 and February 2023 in the city of Petrolina (PE).

### Data analysis

Initially, the database was built by downloading spreadsheet files (.csv) from the SEPLAG/SES/PE database. The database was then organized, and the variables were categorized in binary form, followed by transfer to statistical software and analysis. Microsoft Office Excel 2013 was used to build the database and tables and Stata 14.0 for statistical analysis. Descriptive statistics were then used, using absolute and relative frequency distribution and inferential statistics, using a bivariate analysis. Pearson’s chi-square test and a multiple-analysis model were used.

The associated factors were assessed using binary logistic regression, and the effects were analyzed using the Odds Ratio (OR). The variables were included in the multiple model using the stepwise method, which is a way of automating the best fit of the regression. To this end, the inclusion criterion for the variable in the model was p < 0.20. To control for the different effects of the time used in the analysis period on the probability of death among CVD patients, year dummies were created and inserted into the final multiple model. A significance level of 5% and 95% confidence were adopted.

### Ethical aspects

This study used aggregated data in the public domain, i.e. it followed the ethical precepts established in National Health Council Resolution 510/2016, without the need for evaluation by the Research Ethics Committee (REC).

## Results

Of the 6,704 cases analyzed in the study, 68.1% died and 31.9% recovered. Hospitalization was also required in 93.7% of cases. In terms of demographic characteristics, there was a predominance of females (50.6%), elderly patients (71.1%), and patients of non-white race/color (71.0%). The majority of patients had multiple comorbidities associated with CVD (60.3%), and diabetes was indicated as the main one (44.8%).

By analyzing the demographic and clinical characteristics of patients with CVDs affected by COVID-19, according to the evolution of the case (Table 1), it was possible to see a higher proportion of deaths among non-white women (67.1%), older people (77.9%) and individuals with multiple comorbidities (63.0%).

Although a lower proportion of patients with associated diabetes (46.8%) and chronic kidney disease (5.6%) died, this percentage was higher compared to those who recovered (4.5% and 3.4%, respectively). Among the patients who died, a higher proportion were hospitalized (91.7%).

**Table 1 -:** Demographic and clinical characteristics of patients with CVD affected by COVID-19, according to case evolution (N* = 6,704). Petrolina, PE, Brazil, 2020-2022

**Variable**	**Recovery**		**Death**		**Total**	
	n*	**%** ^†^	n*	**%** ^†^	n*	**%** ^†^
Sex						
Female	1095	51.1	2299	50.4	3394	50.6
Male	1047	48.9	2263	49.6	3310	49.4
Race/color ^‡^						
Non-white	1698	79.3	3063	67.1	4761	71.0
White	444	20.7	1499	32.9	1943	29.0
Age group ^‡^						
Not elderly	930	43.4	1010	22.1	1940	28.9
Elderly	1212	56.6	3552	77.9	4764	71.1
Presence of multiple comorbidities ^‡^						
No	974	45.5	1690	37.1	2664	39.7
Yes	1168	54.5	2872	63.0	4040	60.3
Associated diabetes ^‡^						
No	1274	59.5	2426	53.2	3700	55.2
Yes	868	40.5	2136	46.8	3004	44.8
Associated chronic kidney disease ^‡^						
No	2069	96.6	4309	94.5	6378	95.1
Yes	73	3.4	253	5.6	326	4.9
Associated overweight/obesity						
No	1944	90.8	4107	90.0	6051	90.3
Yes	198	9.2	455	10.0	653	9.7
Associated respiratory diseases						
No	1992	93.0	4208	92.2	6200	92.5
Yes	150	7.0	354	7.8	504	7.5
Need for hospitalization ^‡^						
No	44	2.1	380	8.3	424	6.3
Yes	2098	98.0	4182	91.7	6280	93.7
Case outcome						
Death					4562	68.1
Recovery					2142	32.0

*n = Sample; ^†^% = Percentage; ^‡^Variables that showed a statistically significant difference by Pearson’s chi-square test

When analyzing the factors associated with death as a clinical outcome, it was found that male patients (OR 1.13; p-value = 0.028) who declared themselves white (OR 1.71; p-value = 0.000) and had multiple comorbidities associated with CVD (OR 1.70; p-value = 0.000) had the highest chances of dying. Concerning age group, the chances of death were twice as high in elderly patients (OR 2.57; p-value = 0.000). In addition, the odds of death were lower when hospitalization occurred (OR 0.24; p-value = 0.000), as was the presence of diabetes in the clinical picture (OR 0.83; p-value = 0.038). The comorbidities of respiratory diseases and overweight/obesity were not associated with death, as shown in Table 2.

**Table 2 -:** Multiple adjusted binary logistic regression model of demographic and clinical variables associated with death as the clinical outcome. Petrolina, PE, Brazil, 2020-2022

	Odds Ratio *	**p-value**	**95% CI** ^†^	
Age group				
Elderly	2.57	0.000	2.29	2.88
Non-elderly	1.00			
Sex				
Male	1.13	0.028	1.01	1.26
Female	1.00			
Race/color				
White	1.71	0.000	1.51	1.94
Non-white	1.00			
Multiple comorbidities				
Yes	1.70	0.000	1.42	2.03
No	1.00			
Associated diabetes				
Yes	0.83	0.038	0.70	0.99
No	1.00			
Associated respiratory diseases				
Yes	0.77	0.048	0.59	1.00
No	1.00			
Associated overweight/obesity				
Yes	1.01	0.913	0.81	1.27
No	1.00			
Hospitalization				
Yes	0.24	0.000	0.17	0.33
No	1.00			
Year of notification ^‡^				
2021	0.74	0.000	0.66	0.82
2022	0.85	0.128	0.69	1.05

*Odds Ratio = Probability Ratios; ^†^IC 95% = 95% Confidence Interval; ^‡^Reference category for year dummies = 2020

## Discussion

From the results, it was possible to see that most patients with CVD who were affected by COVID-19 had death as a clinical outcome. Evidence shows that the severity of the infection is associated with a systemic inflammatory response that affects the entire cardiovascular system and increases mortality rates in patients with CVD or its risk factors ^( 3 )^.

The literature indicates that patients with underlying CVD who were infected with SARS-CoV-2 had a worse prognosis due to the damage to the myocardium caused by the virus’s mechanism of action in the cardiovascular system ^( 2 )^. Similarly, a cohort study carried out in Wuhan/China, with 416 patients, showed a statistically significant association between cardiac complications and mortality in patients with COVID-19 ^( 8 )^. This finding demonstrates the clinical vulnerability and high mortality risk of this group.

Regarding the characteristics of the patients in this study, females were more frequent, and non-white race/color prevailed. This profile was similar to that found in a Brazilian ecological study, in which 54.08% of cases were female and 59.85% self-declared as brown ^( 18 )^. Similarly, the prevalence of non-white ethnicity was also observed in North American studies ^( 19 - 20 )^, which found that the majority of patients affected by SARS-CoV-2 infection were of African-American ethnicity.

However, although a higher proportion of deaths was observed in non-white people, it was self-declared white patients who were more likely to die in this study. This result diverges from a study carried out with the records of 7,868 patients with CVD and COVID-19, which analyzed the differences in the prevalence of infection between races and ethnicities and the possible association with case mortality, and showed that African-American/Black and Hispanic populations recorded higher rates of SARS-CoV-2 infection and COVID-19-related mortality ^( 21 )^. Similarly, a study of more than 17 million patients in England found that black people and South Asians had the highest risk of death compared to white people ^( 10 )^.

As for the deaths of patients with CVD and COVID-19, in this study men were more likely to die than women. The high rate of mortality from COVID-19 in men with CVD is widely reported with clinical evidence in the literature ^( 11 - 12 )^. A retrospective multicenter study carried out in Japan with 693 patients with COVID-19 who had CVD showed that male sex was an independent predictor of in-hospital mortality and that, especially among older patients, men had higher in-hospital mortality than women ^( 11 )^.

Some hypotheses have been studied to justify this constant sex difference in the mortality rate, and genetic factors of the X chromosome and sex hormones are considered responsible for the potential protective effects in female patients with COVID-19 ^( 22 )^. These factors allow women to have an enhanced innate immune response, which results in a greater resolution of inflammatory events compared to men ^( 23 )^. In this sense, male patients with COVID-19 are more symptomatic and have the highest disease severity, the highest complication rates, and, ultimately, the highest mortality rates ^( 8 , 12 )^.

Patients with multiple comorbidities associated with CVD and who were considered elderly accounted for most cases in the study and had the highest mortality, which increased the likelihood of death. The presence of comorbidities such as hypertension, diabetes mellitus, and other CVDs can interfere with the severity of the infection and the evolution of the case ^( 13 , 20 )^. In addition, the literature shows that the presence of one or more comorbidities, as well as contributing to the severity of cases, consequently increases the chances of death ^( 7 , 19 )^.

Diabetes was the most prevalent comorbidity among CVD patients reported with COVID-19 in this study, but it decreased the chances of the patient dying, while the presence of overweight or obesity was not significant in the analysis. In Mexico, a study looked at the most frequent cardiac and metabolic comorbidities associated with COVID-19 and found a prevalence of hypertension, followed by diabetes and obesity in hospitalized patients ^( 24 )^. A systematic review and meta-analysis showed that patients with COVID-19 who were at cardiovascular risk due to diabetes, hypertension, and obesity had 1.54, 1.42, and 1.45 times higher risk of mortality, respectively ^( 25 )^, which is different from what was found in this study.

There was a need for hospitalization in the vast majority of the cases analyzed, and this fact is justified by the profile of the patients who, because they have previous comorbidities and advanced age, have severe symptoms that require hospital care and generally need intensive care ^( 8 )^. In line with this reality, an American study carried out in Los Angeles/California found that almost half of the patients (48%) required hospitalization, 36% of whom needed intensive care ^( 20 )^.

However, although this profile of patients has a high rate of hospital mortality, as shown in the literature ^( 3 , 8 , 11 , 25 )^, in this study, the occurrence of hospitalization was statistically significant and showed a lower chance of death. A multicenter study carried out in 73 ICUs, which included 4,198 critically ill patients, showed that the provision of intensive support, especially early intubation in the first 24 hours after ICU admission, was associated with a protective factor and presented the lowest risk of mortality compared to late intubation ^( 26 )^.

In addition, it is worth highlighting the implications of the COVID-19 pandemic on health and CVD monitoring in health services, as there has been a significant and abrupt reduction in cardiac diagnostic procedures in Latin America related to the initial social distancing measures ^( 27 )^. In addition, the interruption or postponement of tests and consultations led to the neglect of complaints potentially related to cardiological conditions, increasing the risk of a serious cardiovascular event, due to the lack of early care caused by fear of contagion or changes in the functioning of health services ^( 28 )^.

For this reason, it is necessary to organize flows and structures compatible with the care profile of patients with CVD at the different levels and health services, especially prioritizing primary care in health promotion and maintenance during pandemic periods, as this has a direct impact on the evolution of critical conditions at the other levels of care ^( 28 )^. In this sense, nurses are among the professionals in the multi-professional team who play a fundamental role in CVD prevention, as they can identify risk factors and social determinants during anamnesis and generate information that can help with multidisciplinary care ^( 29 )^.

The study’s limitations refer to the use of secondary sources, which could show underreporting due to the need for social isolation during the pandemic period, inconsistencies, and incompleteness due to inadequate filling in notification forms, especially in the epidemiological data part. In addition, the collection of information on computerized bases generally involves manual recording on printed forms, with subsequent insertion into the virtual system, which can lead to integrity problems or loss of information and make it impossible to analyze the real number of cases in the state. Another limitation was the large number of comorbidities associated with underlying CVDs, which generated a variety of combinations, making it necessary to choose the most frequent ones to be studied in association or not.

It is worth noting that this study made it possible to identify the epidemiological profile with the greatest potential for worsening and progression to death due to exposure to risk factors that are associated with case mortality and that this profile found in the state of Pernambuco is consistent with the findings in the literature. These findings underpin the vulnerability of the CVD population to SARS-CoV-2 infection and can, therefore, provide support for public policies that prioritize health promotion and infection prevention for this vulnerable group to avoid future high mortality rates.

## Conclusion

This study showed a higher prevalence of female patients in the elderly age group, of non-white race/color and with multiple comorbidities, with diabetes as the main comorbidity found among patients with CVD. Male, elderly, self-declared white patients with multiple comorbidities were more likely to die and accounted for the highest mortality rate. On the other hand, patients with diabetes were less likely to die, and hospitalization was a protective factor, with a lower chance of death.
