# Supplementary material for: Factors associated with mortality in patients with cardiovascular diseases affected by COVID-19: a cross-sectional study
Source: Rev Lat Am Enfermagem. 2025 May 19;33:e4551. doi: 10.1590/1518-8345.7609.4551 (PMC12088660; doi:10.1590/1518-8345.7609.4551)
Supplement: Translation

# Translation: Fatores associados à mortalidade de pacientes com doenças cardiovasculares acometidos pela COVID-19: estudo transversal

**DOI:** 10.1590/1518-8345.7609.4552

**Authors:** Mariane Cardoso Carvalho, Flávia Emília Cavalcante Valença Fernandes, Matheus Vargas dos Santos Almeida, Joebson Maurilio Alves dos Santos, Simone Coelho Amestoy, Rosana Alves de Melo

**Affiliations:** 1 Universidade de Pernambuco, Campus Petrolina, Petrolina, PE, Brasil; 2 Bolsista do Conselho Nacional de Desenvolvimento Científico e Tecnológico (CNPq), Brasil; 3 Universidade de Pernambuco, Faculdade de Administração e Direito de Pernambuco, Recife, PE, Brasil; 4 Universidade Federal do Vale do São Francisco, Departamento de Enfermagem, Petrolina, PE, Brasil

**Keywords:** COVID-19, Mortalidade, Doenças Cardiovasculares, Fatores de Risco, Hospitalização, Notificação

## Abstract

analisar os fatores associados à mortalidade de pacientes com doenças cardiovasculares acometidos pela *Coronavírus Disease-2019*.

estudo transversal, realizado com os dados do acompanhamento das notificações durante a pandemia. Incluíram-se na amostra os casos que possuíam alguma comorbidade cardiovascular e tinham o desfecho clínico. A variável dependente foi a evolução do caso para o óbito. As associações foram testadas pelo método de regressão logística binária*,* por meio da razão de chances (*Odds Ratio*).

prevaleceu o sexo feminino (50,6%), idosos (71,1%), autodeclarados não brancos (71,0%), com múltiplas comorbidades associadas à doença cardiovascular (60,3%), sendo a diabetes a principal delas (44,8%). O estudo sugere que os pacientes homens (OR 1,13; p = 0,028), idosos (OR 2,57; p = 0,000), que se autodeclaram brancos (OR 1,71; p = 0,000) e que possuíam múltiplas comorbidades (OR 1,70; p = 0,000) estavam associados a uma maior chance de evolução para o óbito.

os fatores associados à maior chance de óbito estavam relacionados ao sexo, ao grupo etário e à presença de comorbidades, o que evidenciou a vulnerabilidade dessa população em relação à infecção.

## Destaques

**(1)** Identificação do perfil clínico e demográfico dos pacientes com doenças cardiovasculares acometidos pela Covid-19. **(2)** Os fatores associados à mortalidade foram o sexo, o grupo etário, a raça/cor e as comorbidades. **(3)** Os homens idosos autodeclarados brancos tiveram maior chance de óbito. **(4)** A presença de múltiplas comorbidades associadas às doenças cardiovasculares aumentou a chance de óbito. **(5)** A hospitalização esteve associada a menor chance de óbitos dos casos.

## Introdução

As Doenças Cardiovasculares (DCV) são a principal causa de morbidade e mortalidade no mundo, e representam um risco para a população, pois estima-se que, em 2019, 17,9 milhões de pessoa morreram em decorrência delas, o equivalente a 32% de todas as mortes globais ^( 1 )^. O *Severe Acute Respiratory Syndrome Coronavirus 2* (SARS-CoV-2), que desencadeia a *Coronavirus Disease-2019* (COVID-19), está diretamente associado ao sistema cardiovascular, uma vez que o vírus infecta as células hospedeiras por meio dos receptores da enzima conversora de angiotensina 2 (ECA2) presente em diversos órgãos. A ECA2 se expressa especialmente no coração e nas células epiteliais alveolares dos pulmões ^( 2 - 4 )^.

A ligação da proteína *Spike* do SARS-CoV-2 à proteína ECA2 é seguida pela endocitose do vírus e subsequentemente pela replicação viral, o que causa uma regulação negativa da ECA2 ^( 3 )^ e resulta em sintomas respiratórios que se tornam mais graves na presença de DCV, pois a ECA2 é altamente expressa nesses pacientes em comparação com os indivíduos saudáveis ^( 2 , 4 )^. Com isso, as manifestações clínicas cardiovasculares da COVID-19 incluem principalmente lesão cardíaca aguda, infarto agudo do miocárdio, miocardite, arritmia, insuficiência cardíaca, tromboembolismo venoso e/ou pulmonar e choque ^( 5 )^.

Ademais, em pacientes com hipertensão, doença coronariana e outras cardiomiopatias, a doença viral pode danificar ainda mais as células miocárdicas por meio de dano direto causado pelo vírus, respostas inflamatórias sistêmicas, placa coronária desestabilizada e hipóxia agravada. Estes aspectos tornam estes pacientes mais propensos a sofrer lesão miocárdica após infecção pela COVID-19 e maior risco de morte ^( 6 )^. Por isso, os pacientes com DCV subjacentes, especialmente os idosos, são suscetíveis a maiores riscos de desfechos adversos e à morte, durante as respostas inflamatórias graves da COVID-19, do que os indivíduos mais jovens e saudáveis ^( 7 )^.

A lesão cardíaca é uma condição comum entre os pacientes hospitalizados com COVID-19 e está associada a maior risco de mortalidade intra-hospitalar, mais especificamente em pacientes com DCV preexistentes, pois, segundo uma pesquisa realizada em Wuhan/China, aproximadamente 30% e 60% dos pacientes que tiveram lesões cardíacas possuíam o histórico de doença coronariana e de hipertensão, respectivamente ^( 8 )^.

Da mesma forma, um estudo brasileiro realizado no Rio de Janeiro evidenciou que a incidência de injúria miocárdica de pacientes internados em Unidade de Terapia Intensiva (UTI) com diagnóstico confirmado de COVID-19 foi de 36% da amostra, na qual a hipertensão arterial sistêmica e o Índice de Massa Corpórea (IMC) foram preditores de risco independentes ^( 9 )^.

Os diversos estudos realizados para identificar as características clínicas e/ou demográficas que podem estar associadas à mortalidade desses pacientes evidenciaram a influência de características demográficas (idade, sexo e etnia) ^( 10 - 11 )^ e clínicos (presença de comorbidades) ^( 12 - 13 )^, como fatores de risco para a maior gravidade e mortalidade dos casos. No Reino Unido, um estudo de coorte observacional, realizado com 20.133 pacientes, mostrou que fatores como o sexo masculino, a idade avançada e a presença de comorbidades foram fortes preditores de mortalidade intra-hospitalar ^( 14 )^.

Desse modo, ao se considerar a emergência sanitária que o SARS-CoV-2 representa e as implicações causadas em pacientes com DCV, é necessária a realização de estudos voltados à identificação dos fatores associados à mortalidade da COVID-19 neste grupo vulnerável para a produção de conhecimento científico, uma vez que esse conhecimento pode auxiliar na realização de uma triagem adequada dos pacientes de risco, além de possibilitar a criação de subsídios para a adoção de medidas e estratégias voltadas para o melhor controle da doença e prevenção do agravo.

Dessa forma, o objetivo deste estudo é analisar os fatores associados à mortalidade de pacientes com DCV acometidos pela COVID-19.

## Método

### Delineamento do estudo

Trata-se de um estudo analítico do tipo transversal, realizado com a utilização da base de dados referente ao acompanhamento das notificações por COVID-19 no estado de Pernambuco.

### Local do estudo

O estudo foi realizado no estado de Pernambuco, um estado brasileiro localizado na Região Nordeste do país, com área territorial de 98.067,877 km² e população estimada de 9.674.793 pessoas em 2021 ^( 15 )^.

### População e amostra

A população do estudo foi composta dos casos confirmados de COVID-19 notificados no estado de Pernambuco no período de março de 2020 a dezembro de 2022, com pacientes que apresentavam alguma comorbidade cardiovascular registrada na notificação. Totalizou-se a quantidade de 17.522 casos. Foram incluídos na amostra os casos que possuíam informações sobre o desfecho clínico, isto é, a evolução do caso para óbito ou recuperação. Adotou-se como critério de exclusão os casos que apresentaram ausência de dados ou inconsistência no registro de informações clínicas e demográficas. Com base nesses critérios, a amostra final foi de 6.704 pacientes.

### Variáveis do estudo

A variável dependente foi o óbito como desfecho clínico, enquanto as variáveis independentes foram: sexo (masculino/feminino), grupo etário (idoso ou não idoso), raça/cor (branca e não branca), presença de múltiplas comorbidades (sim/não), classificação dessas comorbidades que estavam associadas ou não às DCV prévias, como a diabetes associada (sim/não), doenças renais crônicas associadas (sim/não), doenças respiratórias associadas (sim/não), sobrepeso/obesidade associado (sim/não) e, por último, a necessidade de hospitalização (sim/não).

Foram considerados como idosos os pacientes que possuíam 60 anos ou mais, conforme o Estatuto da Pessoa Idosa ^( 16 )^, e como não brancos todos os casos em que os pacientes se autodeclararam pretos, pardos, amarelos ou indígenas.

### Coleta dos dados

As informações foram coletadas por meio da plataforma “COVID EM DADOS”, uma base de dados referente ao acompanhamento das notificações por COVID-19 no estado de Pernambuco, disponibilizada pela Secretaria de Planejamento e Gestão (SEPLAG), em conjunto com a Secretaria Estadual de Saúde (SES) e a Agência Estadual de Tecnologia da Informação (ATI) de Pernambuco ^( 17 )^. A coleta e a construção do banco de dados ocorreram entre os meses de dezembro de 2022 a fevereiro de 2023, na cidade de Petrolina (PE).

### Análise dos dados

Inicialmente, foi realizada a construção do banco a partir do *download* de arquivos de planilha (.csv) da base da SEPLAG/SES/PE. Após isso, efetuou-se a organização do banco e a categorização das variáveis de forma binária, com a transferência para o *software* estatístico e a análise em seguida. Foi utilizado o *Microsoft Office Excel* 2013 para a construção do banco de dados e tabelas e o *software* Stata 14.0 para a análise estatística. Em seguida, foi utilizada a estatística descritiva, por meio da distribuição de frequência absoluta e relativa, e a estatística inferencial, por meio de uma análise bivariada. Para tal, utilizou-se o teste Qui-quadrado de Pearson e um modelo múltiplo de análise.

Os fatores associados foram avaliados por meio de regressão logística binária*,* cuja análise dos efeitos foi realizada pela razão de chances (*Odds Ratio* - OR). A inclusão das variáveis no modelo múltiplo se deu pelo método *stepwise*, que representa uma maneira de automatizar o melhor ajuste da regressão. Para isso, adotou-se como critério de inclusão a variável no modelo p < 0,20. Como forma de controlar os diferentes efeitos do tempo utilizado no período de análise sobre a probabilidade de evolução ao óbito entre os pacientes com DCV, foram criadas variáveis *dummies* de ano e inseridas no modelo múltiplo final. Foi adotado o nível de significância de 5% e confiança de 95%.

### Aspectos éticos

Este trabalho utilizou-se de dados agregados e de domínio público, seguindo os preceitos éticos estabelecidos na Resolução do Conselho Nacional de Saúde nº 510/2016, sem a necessidade de avaliação pelo Comitê de Ética em Pesquisa (CEP).

## Resultados

Dos 6.704 casos analisados no estudo, 68,1% evoluíram para o óbito e 31,9% para a recuperação. Ainda, houve a necessidade de hospitalização em 93,7% dos casos. Em relação às características demográficas, predominaram o sexo feminino (50,6%), os pacientes idosos (71,1%) e de raça/cor autodeclarada não branca (71,0%). A maioria dos pacientes possuía múltiplas comorbidades associadas às DCV (60,3%), e a diabetes foi indicada como a principal delas (44,8%).

Por meio da análise das características demográficas e clínicas dos pacientes com DCV acometidos pela COVID-19, segundo a evolução do caso (Tabela 1), foi possível perceber maior proporção do óbito em mulheres não brancas (67,1%), em pessoas idosas (77,9%) e indivíduos com múltiplas comorbidades (63,0%).

Apesar de uma menor proporção de pacientes com diabetes (46,8%) e doença renal crônica (5,6%) associadas terem evoluído a óbito, esse percentual foi maior em comparação aos que apresentaram recuperação (4,5% e 3,4%, respectivamente). Dentre os pacientes que evoluíram para óbito, observou-se uma maior proporção entre aqueles que foram hospitalizados (91,7%).

**Tabela 1 -:** Características demográficas e clínicas dos pacientes com DCV acometidos pela COVID-19, segundo evolução do caso (N* = 6.704). Petrolina, PE, Brasil, 2020-2022

**Variável**	**Recuperação**		**Óbito**		**Total**	
	n*	**%** ^†^	n*	**%** ^†^	n*	**%** ^†^
Sexo						
Feminino	1095	51,1	2299	50,4	3394	50,6
Masculino	1047	48,9	2263	49,6	3310	49,4
Raça/cor ^‡^						
Não branca	1698	79,3	3063	67,1	4761	71,0
Branca	444	20,7	1499	32,9	1943	29,0
Grupo etário ^‡^						
Não idoso	930	43,4	1010	22,1	1940	28,9
Idoso	1212	56,6	3552	77,9	4764	71,1
Presença de múltiplas comorbidades ^‡^						
Não	974	45,5	1690	37,1	2664	39,7
Sim	1168	54,5	2872	63,0	4040	60,3
Diabetes associada ^‡^						
Não	1274	59,5	2426	53,2	3700	55,2
Sim	868	40,5	2136	46,8	3004	44,8
Doenças renais crônicas associadas ^‡^						
Não	2069	96,6	4309	94,5	6378	95,1
Sim	73	3,4	253	5,6	326	4,9
Sobrepeso/obesidade associado						
Não	1944	90,8	4107	90,0	6051	90,3
Sim	198	9,2	455	10,0	653	9,7
Doenças respiratórias associadas						
Não	1992	93,0	4208	92,2	6200	92,5
Sim	150	7,0	354	7,8	504	7,5
Necessidade de hospitalização ^‡^						
Não	44	2,1	380	8,3	424	6,3
Sim	2098	98,0	4182	91,7	6280	93,7
Desfecho do caso						
Óbito					4562	68,1
Recuperação					2142	32,0

*n = Amostra; ^†^% = Porcentagem; ^‡^Variáveis que apresentaram diferença estatisticamente significante pelo teste Qui-quadrado de Pearson

Ao analisar os fatores associados ao óbito como desfecho clínico, verificou-se que os pacientes do sexo masculino (OR 1,13; p-valor = 0,028), que se autodeclaram brancos (OR 1,71; p-valor = 0,000) e possuíam múltiplas comorbidades associadas às DCV (OR 1,70; p-valor = 0,000) indicaram as maiores chances de evoluírem para óbito. Em relação ao grupo etário, as chances de óbito foram duas vezes maiores em pacientes idosos (OR 2,57; p-valor = 0,000). Além disso, observou-se que, na ocorrência de hospitalização, as chances de óbito foram menores (OR 0,24; p-valor = 0,000), assim como a presença de diabetes no quadro clínico (OR 0,83; p-valor = 0,038). As comorbidades doenças respiratórias e sobrepeso/obesidade não estiveram associadas ao óbito, conforme apresentado na Tabela 2.

**Tabela 2 -:** Modelo múltiplo ajustado da regressão logística binária das variáveis demográficas e clínicas associadas ao óbito como desfecho clínico. Petrolina, PE, Brasil, 2020-2022

	***Odds Ratio*** *	**p-valor**	**IC 95%** ^†^	
Grupo etário				
Idoso	2,57	0,000	2,29	2,88
Não idoso	1,00			
Sexo				
Masculino	1,13	0,028	1,01	1,26
Feminino	1,00			
Raça/cor				
Branca	1,71	0,000	1,51	1,94
Não branca	1,00			
Múltiplas comorbidades				
Sim	1,70	0,000	1,42	2,03
Não	1,00			
Diabetes associada				
Sim	0,83	0,038	0,70	0,99
Não	1,00			
Doenças respiratórias associadas				
Sim	0,77	0,048	0,59	1,00
Não	1,00			
Sobrepeso/obesidade associado				
Sim	1,01	0,913	0,81	1,27
Não	1,00			
Hospitalização				
Sim	0,24	0,000	0,17	0,33
Não	1,00			
Ano de notificação ^‡^				
2021	0,74	0,000	0,66	0,82
2022	0,85	0,128	0,69	1,05

*Odds Ratio = Razões de Chance; ^†^IC 95% = Intervalo de Confiança de 95%; ^‡^Categoria de referência para as *dummies* dos anos = 2020

## Discussão

A partir dos resultados, foi possível evidenciar que a maioria dos pacientes com DCV acometida pela COVID-19 teve o óbito como desfecho clínico. Evidências mostram que a gravidade da infecção está associada a uma resposta inflamatória sistêmica que afeta todo o sistema cardiovascular e aumenta as taxas de mortalidade em pacientes com DCV ou seus fatores de risco ^( 3 )^.

A literatura aponta que os pacientes com DCV subjacente, que foram infectados por SARS-CoV-2, tiveram um pior prognóstico devido aos danos no miocárdio causados pelo mecanismo de ação do vírus no sistema cardiovascular ^( 2 )^. Da mesma forma, um estudo de coorte realizado em Wuhan/China, com 416 pacientes, evidenciou a associação estatisticamente significativa entre complicações cardíacas e mortalidade em pacientes com COVID-19 ^( 8 )^. Esse achado demonstra a vulnerabilidade clínica e o alto risco de mortalidade que esse grupo possui.

Em relação às características dos pacientes deste estudo, o sexo feminino foi mais frequente e prevaleceu a raça/cor não branca. Esse perfil foi semelhante ao encontrado em um estudo ecológico brasileiro, em que 54,08% dos casos eram do sexo feminino e 59,85% se autodeclaravam pardos ^( 18 )^. Da mesma forma, a prevalência de etnia não branca também foi observada em pesquisas norte-americanas ^( 19 - 20 )^, que observaram que a maioria dos pacientes acometidos pela infecção do SARS-CoV-2 era de etnia afro-americana.

No entanto, apesar de ter sido observada maior proporção de óbitos em pessoas não brancas, foram os pacientes autodeclarados brancos que apresentaram maior chance de ir a óbito neste estudo. Esse resultado diverge de um estudo realizado com os registros de 7.868 pacientes com DCV e COVID-19, que analisou as diferenças na prevalência de infecção entre as raças e as etnias e a possível associação com a mortalidade dos casos, e evidenciou que as populações afro-americanas/negras e hispânicas registaram taxas mais elevadas de infecção por SARS-CoV-2 e de mortalidade relacionada com a COVID-19 ^( 21 )^. Da mesma forma, um estudo com dados de mais de 17 milhões de pacientes, realizado na Inglaterra, identificou que negros e sul-asiáticos apresentaram maior risco de morte quando comparados às pessoas de etnia branca ^( 10 )^.

Em relação aos óbitos dos pacientes com DCV e COVID-19, nesta pesquisa os homens tiveram uma maior chance de evoluir a óbito quando comparados às mulheres. O alto índice de mortalidade por COVID-19 em homens com DCV é amplamente relatado por evidências clínicas na literatura ^( 11 - 12 )^. Um estudo retrospectivo multicêntrico realizado no Japão, com 693 pacientes com COVID-19 e DCV, demonstrou que o sexo masculino era um preditor independente de mortalidade intra-hospitalar e que, especialmente entre os pacientes mais velhos, os homens tiveram maior mortalidade hospitalar do que as mulheres ^( 11 )^.

Algumas hipóteses foram estudadas para justificar essa constante diferença de sexo no índice de mortalidade, e os fatores genéticos do cromossomo X e os hormônios sexuais são considerados responsáveis pelos potenciais efeitos protetores em pacientes do sexo feminino com COVID-19 ^( 22 )^. Esses fatores permitem que as mulheres tenham uma resposta imune inata aprimorada, o que resulta em maior resolução de eventos inflamatórios em comparação com os homens ^( 23 )^. Nesse sentido, os pacientes do sexo masculino com COVID-19 são mais sintomáticos e apresentam maior gravidade da doença, maiores taxas de complicações e, em última análise, maior mortalidade ^( 8 , 12 )^.

Os pacientes que possuíam múltiplas comorbidades associadas às DCV e que eram considerados idosos representaram a maioria dos casos no estudo e apresentaram maior mortalidade, o que aumentou a probabilidade de evolução para óbito. A presença de comorbidades como hipertensão, *Diabetes Mellitus* e outras DCV podem interferir na gravidade da infecção e na evolução do caso ^( 13 , 20 )^. Além disso, a literatura aponta que a presença de uma ou mais comorbidades, além de contribuir para a gravidade dos casos, consequentemente aumenta as chances de óbito ^( 7 , 19 )^.

O diabetes foi a comorbidade de maior prevalência entre os pacientes com DCV notificados com COVID-19 neste estudo, porém, diminuiu as chances de o paciente evoluir para óbito, ao passo que a presença de sobrepeso ou obesidade não foi significante na análise. No México, um estudo observou as comorbidades cardíacas e metabólicas mais frequentes associadas à COVID-19 e evidenciou a prevalência de hipertensão, seguida de diabetes e obesidade em pacientes hospitalizados ^( 24 )^. Uma revisão sistemática e uma metanálise evidenciaram que os pacientes com COVID-19 que possuíam risco cardiovascular, devido à presença de diabetes, hipertensão e obesidade, tiveram um risco de mortalidade 1,54 vezes, 1,42 vezes, 1,45 vezes maior, respectivamente ^( 25 )^, o que indicou divergência dos achados desta pesquisa.

Houve necessidade de hospitalização na grande maioria dos casos analisados, e esse fato é justificado pelo próprio perfil dos pacientes que, por possuírem comorbidades prévias e idade avançada, apresentam sintomas graves que necessitam de assistência hospitalar e, geralmente, precisam de cuidados intensivos ^( 8 )^. De forma semelhante a essa realidade, um estudo norte-americano realizado em Los Angeles, na Califórnia, observou que quase metade dos pacientes (48%) necessitou de internação hospitalar, dos quais 36% necessitaram de cuidados intensivos ^( 20 )^.

Entretanto, apesar desse perfil de pacientes apresentar uma taxa elevada de mortalidade hospitalar, conforme apresentado na literatura ^( 3 , 8 , 11 , 25 )^, neste estudo a ocorrência da hospitalização teve significância estatística e apresentou uma menor chance de óbito. Um estudo multicêntrico realizado em 73 UTIs, e que incluiu 4.198 pacientes graves, mostrou que a disposição de suporte intensivo, especialmente a intubação precoce nas primeiras 24 horas após a admissão na UTI, foi associada a um fator protetor e apresentou o menor risco de mortalidade em comparação com a realização da intubação tardia ^( 26 )^.

Além disso, vale ressaltar as implicações da pandemia da COVID-19 no acompanhamento de saúde e das DCV em serviços de saúde, pois houve uma redução significativa e abrupta de procedimentos diagnósticos cardíacos na América Latina, relacionada às medidas de distanciamento social iniciais ^( 27 )^. Ademais, a interrupção ou aditamento de exames e consultas levou ao negligenciamento de queixas potencialmente relacionadas às condições cardiológicas e elevou o risco de evento cardiovascular grave, devido à falta de atendimento precoce causado pelo medo do contágio ou pelas mudanças no funcionamento dos serviços de saúde ^( 28 )^.

Por isso, é necessário organizar fluxos e estruturas compatíveis com o perfil assistencial de pacientes com DCV, nos diferentes níveis e serviços de saúde, especialmente a priorização da assistência primária na promoção e manutenção da saúde em períodos pandêmicos, pois isso impacta diretamente a evolução dos quadros críticos no demais níveis de atenção ^( 28 )^. Nesse sentido, o enfermeiro está entre os profissionais da equipe multiprofissional que possuem importante e fundamental papel na prevenção de DCV, pois ele é capaz de identificar os fatores de riscos e os determinantes sociais durante a anamnese, e gerar as informações que podem auxiliar no cuidado multidisciplinar ^( 29 )^.

As limitações do estudo se referem ao uso de fontes secundárias, que poderiam apresentar subnotificações pela necessidade de isolamento social durante o período pandêmico, inconsistências e incompletudes em razão do preenchimento inadequado das fichas de notificação, principalmente na parte dos dados epidemiológicos. Ademais, a coleta de informações em bases informatizadas, em geral, compreende o registro manual em formulários impressos, com a posterior inserção no sistema virtual, o que pode implicar em problemas de integridade ou perdas de informações e impossibilitar a análise do quantitativo real dos casos ocorridos no estado. Outra limitação foi a grande quantidade de comorbidades associadas às DCV de base, que gerou uma diversidade de combinações, sendo necessário escolher as mais frequentes para serem estudadas de forma associada ou não.

Vale ressaltar que este estudo possibilitou a identificação do perfil epidemiológico com maior potencialidade de agravamento e evolução para óbito em função da exposição aos fatores de risco que estão associados à mortalidade dos casos, e que esse perfil encontrado no estado de Pernambuco é condizente com os achados na literatura. Esses achados fundamentam a vulnerabilidade que a população com DCV possui em relação à infecção do SARS-CoV-2 e, portanto, pode oferecer subsídios para políticas públicas que priorizem a promoção à saúde e a prevenção de infecção desse grupo vulnerável, a fim de evitar futuras taxas de mortalidade elevadas.

## Conclusão

Este estudo permitiu demonstrar uma maior prevalência de pacientes mulheres, do grupo etário idoso, de raça/cor não branca e com múltiplas comorbidades, com a diabetes como a principal comorbidade encontrada entre os pacientes com DCV. Os pacientes do sexo masculino, idosos, autodeclarados brancos e com múltiplas comorbidades apresentaram uma maior chance de evolução para óbito, e representaram a maior mortalidade dentre os casos. Em contrapartida, os pacientes com diabetes tiveram a menor probabilidade de ir a óbito e a hospitalização se constituiu como um fator protetor, com menor chance de óbito.

# Translation: Factores asociados a la mortalidad de pacientes con enfermedades cardiovasculares afectados por la COVID-19: estudio transversal

**DOI:** 10.1590/1518-8345.7609.4550

**Authors:** Mariane Cardoso Carvalho, Flávia Emília Cavalcante Valença Fernandes, Matheus Vargas dos Santos Almeida, Joebson Maurilio Alves dos Santos, Simone Coelho Amestoy, Rosana Alves de Melo

**Affiliations:** 1 Universidade de Pernambuco, Campus Petrolina, Petrolina, PE, Brasil; 2 Becaria del Conselho Nacional de Desenvolvimento Científico e Tecnológico (CNPq), Brasil; 3 Universidade de Pernambuco, Faculdade de Administração e Direito de Pernambuco, Recife, PE, Brasil; 4 Universidade Federal do Vale do São Francisco, Departamento de Enfermagem, Petrolina, PE, Brasil

**Keywords:** COVID-19, Mortalidad, Enfermedades Cardiovasculares, Factores de Riesgo, Hospitalización, Notificación

## Abstract

analizar los factores asociados a la mortalidad de los pacientes con enfermedades cardiovasculares afectados por el *Coronavirus Disease-2019*.

estudio transversal, realizado con datos del seguimiento de las notificaciones durante la pandemia. Se incluyeron en la muestra los casos que presentaban alguna comorbilidad cardiovascular y tenían evolución clínica. La variable dependiente fue la evolución clínica del caso a muerte. Las asociaciones se probaron utilizando el método de regresión logística binaria*,* mediante la razón de probabilidades (*Odds Ratio*).

predominaron el sexo femenino (50,6%), ser adulto mayor (71,1%), los autodeclarados no blancos (71,0%), con múltiples comorbilidades asociadas a la enfermedad cardiovascular (60,3%), de las cuales la principal fue la diabetes (44,8%). El estudio sugiere que los pacientes hombres (OR 1,13; p=0,028), adultos mayores (OR 2,57; p = 0,000), que se autodeclararon blancos (OR 1,71; p = 0,000) y que tenían múltiples comorbilidades (OR 1,70; p = 0,000), se asociaron con una mayor probabilidad de muerte.

los factores asociados a una mayor probabilidad de muerte se relacionaban con el sexo, el grupo etario y la presencia de comorbilidades, lo que demuestra la vulnerabilidad de esta población frente a la infección.

## Destacados

**(1)** Identificar el perfil clínico y demográfico de pacientes con ECV afectados por la COVID-19.**(2)** Los factores asociados a la mortalidad fueron sexo, grupo etario, raza/color y comorbilidades. **(3)** Los hombres adultos mayores que se autodeclararon blancos tenían mayor probabilidad de muerte. **(4)** La presencia de múltiples comorbilidades asociadas a la ECV aumentó la probabilidad de muerte. **(5)** La hospitalización se asoció con una menor probabilidad de muerte.

## Introducción

Las Enfermedades Cardiovasculares (ECV) son la principal causa de morbilidad y mortalidad en el mundo, y representan un riesgo para la población, ya que se estima que 17,9 millones de personas murieron como consecuencia de ella en 2019, lo que equivale al 32% de todas las muertes a nivel mundial ^( 1 )^. El *Severe Acute Respiratory Syndrome Coronavirus 2* (SARS-CoV-2), que desencadena la *Coronavirus Disease-2019* (COVID-19), está directamente asociado al sistema cardiovascular, ya que el virus infecta las células huésped a través de los receptores de la enzima convertidora de angiotensina 2 (ECA2) presente en varios órganos y se expresa altamente, especialmente en el corazón y en las células epiteliales alveolares de los pulmones ^( 2 - 4 )^.

Tras la unión de la proteína *Spike* del SARS-CoV-2 a la proteína ECA2 viene la endocitosis viral y posteriormente la replicación viral, lo que provoca la regulación negativa de la ECA2 ^( 3 )^ y da lugar a síntomas respiratorios que se tornan más graves en presencia de ECV, ya que la ECA2 se expresa altamente en estos pacientes en comparación con individuos sanos ^( 2 , 4 )^. Por lo tanto, las manifestaciones clínicas cardiovasculares de la COVID-19 incluyen principalmente lesión cardiaca aguda, infarto agudo de miocardio, miocarditis, arritmia, insuficiencia cardiaca, tromboembolismo venoso y/o pulmonar y *shock* ^( 5 )^.

Además, en pacientes con hipertensión, enfermedad coronaria y otras miocardiopatías, la enfermedad viral puede dañar aún más las células del miocardio por medio de: daño directo causado por el virus, respuestas inflamatorias sistémicas, placa coronaria desestabilizada e hipoxia agravada. Dichos aspectos hacen que estos pacientes sean más propensos a sufrir lesión miocárdica tras la infección por COVID-19 y tengan mayor riesgo de muerte ^( 6 )^. Por ello, los pacientes con ECV subyacente, especialmente los adultos mayores, tienen mayor riesgo de resultados adversos y muerte durante respuestas inflamatorias graves a la COVID-19 que los individuos más jóvenes y saludables ^( 7 )^.

La lesión cardíaca es una condición común en los pacientes hospitalizados con COVID-19 y se asocia con un mayor riesgo de mortalidad intrahospitalaria, más específicamente en pacientes con ECV preexistente, ya que, según un estudio realizado en Wuhan/China, aproximadamente el 30% y el 60% de los pacientes que presentaron lesiones cardíacas tenían antecedentes de enfermedad coronaria e hipertensión, respectivamente ^( 8 )^.

De manera similar, un estudio brasileño realizado en Río de Janeiro reveló que la incidencia de lesión miocárdica en pacientes ingresados en la Unidad de Cuidados Intensivos (UCI) con diagnóstico confirmado de COVID-19 fue del 36% de la muestra, en la cual la hipertensión arterial sistémica y el índice de masa corporal (IMC) fueron predictores de riesgo independientes ^( 9 )^.

Los diversos estudios realizados para identificar las características clínicas y/o demográficas que pueden estar asociadas a la mortalidad de estos pacientes demuestran la influencia de las características demográficas (edad, sexo y etnia) ^( 10 - 11 )^ y clínicas (presencia de comorbilidades) ^( 12 - 13 )^, así como también los factores de riesgo de mayor gravedad y mortalidad de los casos analizados. En el Reino Unido, un estudio de cohorte observacional con 20.133 pacientes demostró que factores como el sexo masculino, ser adulto mayor y tener comorbilidades eran fuertes predictores de mortalidad intrahospitalaria ^( 14 )^.

Por ende, al considerar la emergencia sanitaria que representa el SARS-CoV-2, y las implicancias que tiene para los pacientes con ECV, es necesario realizar estudios dirigidos a identificar los factores asociados a la mortalidad por COVID-19 en este grupo vulnerable, para producir conocimiento científico, ya que este conocimiento puede ayudar a realizar una detección adecuada de los pacientes en riesgo, además de permitir que se creen herramientas para adoptar medidas y estrategias dirigidas a controlar mejor la enfermedad y a prevenir daños.

Por lo tanto, el objetivo de este estudio es analizar los factores asociados a la mortalidad de los pacientes con ECV afectados por la COVID-19.

## Método

### Diseño del estudio

Se trata de un estudio analítico transversal, realizado utilizando la base de datos sobre el seguimiento de las notificaciones de COVID-19 en el estado de Pernambuco.

### Ubicación del estudio

El estudio se realizó en el estado de Pernambuco, estado brasileño ubicado en la región Nordeste del país, con una superficie territorial de 98.067,877 km² y una población estimada de 9.674.793 personas en 2021 ^( 15 )^.

### Población y muestra

La población del estudio estuvo conformada por casos confirmados de COVID-19 notificados en el estado de Pernambuco, en el período de marzo de 2020 a diciembre de 2022, y que presentaban alguna comorbilidad cardiovascular registrada en la notificación. El número total de casos fue 17.522. Se incluyeron en la muestra los casos que tenían información sobre el desenlace clínico, es decir, la evolución del caso a muerte o recuperación. El criterio de exclusión adoptado fueron los casos que presentaban falta o inconsistencia en el registro de información clínica y demográfica. En base a estos criterios, la muestra final estuvo compuesta por 6.704 pacientes.

### Variables del estudio

La variable dependiente fue la muerte como resultado clínico, mientras que las variables independientes fueron: sexo (masculino/femenino), grupo etario (adulto mayor o no adulto mayor), raza/color (blanco y no blanco), presencia de múltiples comorbilidades (sí/no), clasificación de las comorbilidades que estaban asociadas o no a ECV previa, como diabetes asociada (sí/no), enfermedades renales crónicas asociadas (sí/no), enfermedades respiratorias asociadas (sí/no), sobrepeso/obesidad asociada (sí/no) y, por último, la necesidad de hospitalización (sí/no).

Se consideraron adultos mayores los pacientes que tenían 60 años o más, según el Estatuto de las Personas Mayores ^( 16 )^, y no blancos todos los casos en que los pacientes se autodeclararon negros, mulatos, amarillos o indígenas.

### Recopilación de datos

La información se recolectó de la plataforma “COVID EM DADOS”, base de datos relacionada al seguimiento de las notificaciones de COVID-19 en el estado de Pernambuco, puesta a disposición por la Secretaría de Planificación y Gestión (SEPLAG), en conjunto con la Secretaría Estadual de Salud (SES) y la Agencia Estadual de Tecnología de la Información (ATI) de Pernambuco ^( 17 )^. La recolección y creación de la base de datos se realizó entre diciembre de 2022 y febrero de 2023, en la ciudad de Petrolina (PE).

### Análisis de datos

Inicialmente, la base de datos se creó con los archivos (.csv) descargados de la base de datos SEPLAG/SES/PE. A continuación, se organizó la base y se categorizaron las variables en forma binaria, mediante la transferencia al *software* estadístico y el posterior análisis. Se utilizó *Microsoft Office Excel 2013* para crear la base de datos y las tablas y el *softwareStata* 14.0 para el análisis estadístico. Luego, se utilizó estadística descriptiva, a través de la distribución de frecuencias absolutas y relativas, y estadística inferencial, mediante un análisis bivariado. Para ello se utilizó la prueba Chi-cuadrado de Pearson y un modelo de análisis múltiple*.*

Los factores asociados se evaluaron mediante regresión logística binaria*,* y el análisis de los efectos se realizó mediante la razón de probabilidades (*Odds Ratio,* OR). La inclusión de las variables múltiples en el modelo se realizó mediante el método *stepwise*, el cual representa una forma de automatizar el mejor ajuste de la regresión. Para ello, el criterio adoptado de inclusión de la variable en el modelo fue p < 0,20. Como forma de controlar los diferentes efectos del tiempo utilizado en el período de análisis sobre la probabilidad de muerte en pacientes con ECV, se crearon variables *dummies* de año y se insertaron en el modelo múltiple final. Se adoptó un nivel de significación del 5% y de confianza del 95%.

### Aspectos éticos

Este trabajo utilizó datos agregados y de dominio público, es decir, siguió los preceptos éticos establecidos en la Resolución del Consejo Nacional de Salud brasileño nº 510/2016, por lo que no fue necesario someterlo a la evaluación del Comité de Ética en Investigación.

## Resultados

De los 6.704 casos analizados en el estudio, el desenlace clínico del 68,1% fue la muerte y el desenlace del 31,9% la recuperación. Además, en el 93,7% de los casos fue necesario hospitalizar al paciente. Con respecto a las características demográficas, predominaron los pacientes de sexo femenino (50,6%), adultos mayores (71,1%) y de raza/color no blanco autodeclarado (71,0%). La mayoría de los pacientes presentaba múltiples comorbilidades asociadas a la ECV (60,3%), de las cuales la principal fue la diabetes (44,8%).

A través del análisis de las características demográficas y clínicas de los pacientes con ECV afectados por la COVID-19, según la evolución del caso (Tabla 1), fue posible observar que había una mayor proporción de muertes en mujeres no blancas (67,1%), en adultos mayores (77,9%) y en individuos con múltiples comorbilidades (63,0%).

Si bien la proporción de pacientes con diabetes (46,8%) y enfermedad renal crónica (5,6%) asociadas que evolucionó a muerte fue menor, ese porcentaje fue mayor que el de los que se recuperaron (4,5% y 3,4%, respectivamente). Entre los pacientes que fallecieron, se observó una mayor proporción en los que estuvieron hospitalizados (91,7%).

**Tabla 1 -:** Características demográficas y clínicas de los pacientes con ECV afectados por la COVID-19, según la evolución del caso (N*= 6.704). Petrolina, PE, Brasil, 2020-2022

**Variable**	**Recuperación**		**Muerte**		**Total**	
	n*	**%** ^†^	n*	**%** ^†^	n*	**%** ^†^
Sexo						
Femenino	1095	51,1	2299	50,4	3394	50,6
Masculino	1047	48,9	2263	49,6	3310	49,4
Raza/color ^‡^						
No blanco	1698	79,3	3063	67,1	4761	71,0
Blanco	444	20,7	1499	32,9	1943	29,0
Grupo etario ^‡^						
No adulto mayor	930	43,4	1010	22,1	1940	28,9
Adulto mayor	1212	56,6	3552	77,9	4764	71,1
Presencia de múltiples comorbilidades ^‡^						
No	974	45,5	1690	37,1	2664	39,7
Sí	1168	54,5	2872	63,0	4040	60,3
Diabetes asociada ^‡^						
No	1274	59,5	2426	53,2	3700	55,2
Sí	868	40,5	2136	46,8	3004	44,8
Enfermedades renales crónicas asociadas ^‡^						
No	2069	96,6	4309	94,5	6378	95,1
Sí	73	3,4	253	5,6	326	4,9
Sobrepeso/obesidad asociada						
No	1944	90,8	4107	90,0	6051	90,3
Sí	198	9,2	455	10,0	653	9,7
Enfermedades respiratorias asociadas						
No	1992	93,0	4208	92,2	6200	92,5
Sí	150	7,0	354	7,8	504	7,5
Necesidad de hospitalización ^‡^						
No	44	2,1	380	8,3	424	6,3
Sí	2098	98,0	4182	91,7	6280	93,7
Desenlace del caso						
Muerte					4562	68,1
Recuperación					2142	32,0

*n = Muestra; ^†^% = Porcentaje; ^‡^Variables que presentaron diferencia estadísticamente significativa según la prueba Chi -cuadrado de Pearson

Al analizar los factores asociados a la muerte como desenlace clínico, se encontró que los pacientes del sexo masculino (OR 1,13; p-valor = 0,028), que se autodeclararon blancos (OR 1,71; p-valor = 0,000) y tenían múltiples comorbilidades asociadas a la ECV (OR 1,70; valor de p = 0,000) tenían mayores probabilidades de muerte. En cuanto al grupo etario, las probabilidades de muerte fueron dos veces mayores en los pacientes adultos mayores (OR 2,57; valor de p = 0,000). Además, se observó que, en caso de hospitalización, las probabilidades de muerte fueron menores (OR 0,24; p-valor = 0,000), al igual que la presencia de diabetes en el cuadro clínico (OR 0,83; p-valor = 0,038). Las comorbilidades enfermedades respiratorias y sobrepeso/obesidad no se asociaron con la muerte, como se puede ver en la Tabla 2.

**Tabla 2 -:** Modelo múltiple ajustado de regresión logística binaria de las variables demográficas y clínicas asociadas con la muerte como desenlace clínico. Petrolina, PE, Brasil, 2020-2022

	Odds Ratio*	**valor de p**	**IC 95 %** ^†^	
Grupo etario				
Adulto mayor	2,57	0,000	2,29	2,88
No adulto mayor	1,00			
Sexo				
Masculino	1,13	0,028	1,01	1,26
Femenino	1,00			
Raza/color				
Blanco	1,71	0,000	1,51	1,94
No blanco	1,00			
Comorbilidades múltiples				
Sí	1,70	0,000	1,42	2,03
No	1,00			
Diabetes asociada				
Sí	0,83	0,038	0,70	0,99
No	1,00			
Enfermedades respiratorias asociadas				
Sí	0,77	0,048	0,59	1,00
No	1,00			
Sobrepeso/obesidad asociada				
Sí	1,01	0,913	0,81	1,27
No	1,00			
Hospitalización				
Sí	0,24	0,000	0,17	0,33
No	1,00			
Año de notificación ^‡^				
2021	0,74	0,000	0,66	0,82
2022	0,85	0,128	0,69	1,05

*Odds Ratio = Razón de probabilidades; ^†^IC 95% = Intervalo de confianza del 95%; ^‡^Categoría de referencia para *dummies* de los años = 2020

## Discusión

A partir de los resultados, se pudo demostrar que el resultado clínico de la mayoría de los pacientes con ECV afectados por la COVID-19 fue la muerte. La evidencia muestra que la severidad de la infección está asociada con una respuesta inflamatoria sistémica que afecta a todo el sistema cardiovascular y aumenta las tasas de mortalidad en pacientes con ECV o con factores de riesgo ^( 3 )^.

La literatura indica que los pacientes con ECV subyacente que fueron afectados por SARS-CoV-2 tuvieron un peor pronóstico debido al daño miocárdico causado por el mecanismo de acción del virus en el sistema cardiovascular ^( 2 )^. De manera similar, un estudio de cohorte realizado en Wuhan/China, con 416 pacientes, demostró que había una asociación estadísticamente significativa entre complicaciones cardíacas y mortalidad en pacientes con COVID-19 ^( 8 )^. Este hallazgo demuestra la vulnerabilidad clínica y el alto riesgo de mortalidad que tiene este grupo.

En cuanto a las características de los pacientes de este estudio, el sexo femenino fue más frecuente y predominó la raza/color no blanco. Este perfil fue similar al encontrado en un estudio ecológico brasileño, en el que el 54,08% de los casos eran del sexo femenino y el 59,85% se autodeclaró mulato ^( 18 )^. De manera similar, la prevalencia de etnia no blanca también se observó en estudios norteamericanos ^( 19 - 20 )^, que indicaron que la mayoría de los pacientes afectados por la infección por SARS-CoV-2 eran de etnia afroamericana.

Sin embargo, a pesar de que se observó una mayor proporción de muertes en personas no blancas, fueron los pacientes que se autodeclararon blancos quienes tuvieron una mayor probabilidad de morir en este estudio. Este resultado difiere del que arrojó un estudio realizado con los registros de 7.868 pacientes con ECV y COVID-19, que analizó las diferencias en la prevalencia de infección entre razas y etnias y la posible asociación con la mortalidad de los casos, y reveló que las poblaciones afroamericanas/negras y las mujeres hispanas tuvieron las tasas más altas de infección por SARS-CoV-2 y mortalidad relacionada con la COVID-19 ^( 21 )^. De manera similar, un estudio con datos de más de 17 millones de pacientes realizado en Inglaterra identificó que las personas negras y del sur de Asia tenían mayor riesgo de muerte que las personas de etnia blanca ^( 10 )^.

En lo que respecta a las muertes de pacientes con ECV y COVID-19, en esta investigación los hombres tuvieron mayor probabilidad de muerte que las mujeres. La alta tasa de mortalidad por COVID-19 en hombres con ECV es ampliamente informada con evidencia clínica en la literatura ^( 11 - 12 )^. Un estudio retrospectivo multicéntrico realizado en Japón, con 693 pacientes con COVID-19 y ECV, demostró que el sexo masculino fue un predictor independiente de mortalidad intrahospitalaria y que, especialmente en el caso de los pacientes de mayor edad, los hombres tuvieron mayor mortalidad hospitalaria que las mujeres ^( 11 )^.

Se han estudiado algunas hipótesis para justificar esta diferencia constante de sexo en la tasa de mortalidad, y se considera que los factores genéticos del cromosoma X y las hormonas sexuales son los responsables de los potenciales efectos protectores en pacientes femeninos con COVID-19 ^( 22 )^. Estos factores permiten que las mujeres tengan una respuesta inmune innata mejorada, que hace que tengan una mayor resolución de eventos inflamatorios que los hombres ^( 23 )^. Por ende, los pacientes del sexo masculino con COVID-19 son más sintomáticos y presentan mayor gravedad de la enfermedad, mayores tasas de complicaciones y, en definitiva, mayor mortalidad ^( 8 , 12 )^.

Los pacientes que presentaban múltiples comorbilidades asociadas a ECV y que eran considerados adultos mayores representaban la mayoría de los casos en el estudio y presentaban la mayor mortalidad, lo que aumentó la probabilidad de muerte. La presencia de comorbilidades como hipertensión, *Diabetes Mellitus* y otras ECV pueden interferir en la gravedad de la infección y la evolución del caso ^( 13 , 20 )^. Por otro lado, la literatura indica que la presencia de una o más comorbilidades, además de contribuir a la gravedad del caso, aumenta la probabilidad de muerte ^( 7 , 19 )^.

En este estudio, la diabetes fue la comorbilidad más prevalente en los pacientes con ECV notificados con COVID-19, sin embargo, redujo las probabilidades de muerte, mientras que la presencia de sobrepeso u obesidad no fue significativa en el análisis. En México, un estudio observó las comorbilidades cardíacas y metabólicas más frecuentes asociadas a la COVID-19 y destacó la prevalencia de hipertensión, seguida de diabetes y obesidad en pacientes hospitalizados ^( 24 )^. Una revisión sistemática y un metaanálisis mostró que los pacientes con COVID-19, que tenían riesgo cardiovascular por la presencia de diabetes, hipertensión y obesidad, tenían un riesgo de mortalidad 1,54 veces, 1,42 veces, 1,45 veces mayor, respectivamente ^( 25 )^, lo que difiere de lo observado en esta investigación.

En la gran mayoría de los casos analizados fue necesario hospitalizar a los pacientes, y este hecho se debe al perfil de los mismos que, por tener comorbilidades previas y edad avanzada, presentan cuadros graves que requieren asistencia hospitalaria y, generalmente, requieren cuidados intensivos ^( 8 )^. De forma similar, en un estudio norteamericano realizado en Los Ángeles, California, se observó que fue necesario hospitalizar a casi la mitad de los pacientes (48%), de los cuales el 36% requirió cuidados intensivos ^( 20 )^.

Sin embargo, a pesar de que este perfil de paciente presenta una alta tasa de mortalidad hospitalaria, como lo indica la literatura ^( 3 , 8 , 11 , 25 )^, en este estudio, la hospitalización tuvo significación estadística y presentó menor probabilidad de muerte. Un estudio multicéntrico realizado en 73 UCI, que incluyó a 4.198 pacientes críticos, reveló que brindar soporte intensivo, especialmente la intubación temprana en las primeras 24 horas después del ingreso a la UCI, se asoció con un factor protector y presentó menor riesgo de mortalidad que la intubación diferida ^( 26 )^.

Además, cabe destacar las implicancias que tuvo la pandemia de COVID-19 en el seguimiento de la salud y de las ECV en los servicios de salud, ya que hubo una reducción significativa y abrupta de los procedimientos de diagnóstico cardíaco en América Latina debido a las medidas iniciales de distanciamiento social ^( 27 )^. Por otro lado, la interrupción o postergación de exámenes y consultas provocó la desatención de quejas potencialmente relacionadas con afecciones cardíacas y aumentó el riesgo de eventos cardiovasculares graves, debido a la falta de atención temprana provocada por el miedo al contagio o los cambios en el funcionamiento de los servicios de salud ^( 28 )^.

Por ello, es necesario organizar flujos y estructuras compatibles con el perfil de atención de los pacientes con ECV, en los diferentes niveles y servicios de salud, fundamentalmente priorizar la atención primaria en la promoción y mantenimiento de la salud en períodos de pandemia, ya que esto afecta directamente la evolución de los cuadros críticos en los otros niveles de atención ^( 28 )^. En este sentido, el enfermero se encuentra entre los profesionales del equipo multidisciplinario que tienen un papel importante y fundamental en la prevención de la ECV, dado que puede identificar los factores de riesgo y los determinantes sociales durante la anamnesis, y generar información que puede contribuir a la atención multidisciplinaria ^( 29 )^.

Se consideran limitaciones del estudio el uso de fuentes secundarias, que podrían presentar subregistro debido a la necesidad de aislamiento social durante el período de la pandemia, las inconsistencias y la falta de información, producto de la inadecuada cumplimentación de los formularios de notificación, especialmente en la sección de datos epidemiológicos. Además, la recolección de información en bases de datos informatizadas, por lo general, implica el registro manual en formularios impresos, con posterior inserción en el sistema virtual, lo que puede dar lugar a problemas de integridad o pérdida de información e imposibilitar el análisis de la cantidad real de los casos en el estado. Otra limitación fue el gran número de comorbilidades asociadas a la ECV de base, que generó una diversidad de combinaciones, e hizo que fuera necesario elegir las más frecuentes para estudiar si tenían asociación o no.

Cabe destacar que este estudio permitió identificar el perfil epidemiológico con mayor potencial de agravamiento y evolución a muerte en función de la exposición a factores de riesgo que se asocian a la mortalidad de los casos, y que el perfil encontrado en el estado de Pernambuco es consistente con los hallazgos presentes en la literatura. Estos hallazgos respaldan la vulnerabilidad que tiene la población con ECV a la infección por SARS-CoV-2 y, por lo tanto, pueden contribuir a las políticas públicas que prioricen la promoción de la salud y la prevención de infecciones en este grupo vulnerable, a fin de evitar futuras tasas de mortalidad elevadas.

## Conclusión

Este estudio demostró que hubo una mayor prevalencia de pacientes del sexo femenino, adultos mayores, de raza/color no blanco y con múltiples comorbilidades, de las cuales la diabetes es la principal comorbilidad encontrada en los pacientes con ECV. Los pacientes del sexo masculino, adultos mayores, que se autodeclararon blancos y presentaban múltiples comorbilidades tenían mayor probabilidad de muerte y representaron la tasa de mortalidad más alta en los casos analizados. Por el contrario, los pacientes con diabetes tenían la menor probabilidad de muerte y la hospitalización fue un factor protector, con una menor probabilidad de muerte.
